# Associations of functional alanine-glyoxylate aminotransferase 2 gene variants with atrial fibrillation and ischemic stroke

**DOI:** 10.1038/srep23207

**Published:** 2016-03-17

**Authors:** Ilkka Seppälä, Marcus E. Kleber, Steve Bevan, Leo-Pekka Lyytikäinen, Niku Oksala, Jussi A. Hernesniemi, Kari-Matti Mäkelä, Peter M. Rothwell, Cathie Sudlow, Martin Dichgans, Nina Mononen, Efthymia Vlachopoulou, Juha Sinisalo, Graciela E. Delgado, Reijo Laaksonen, Tuomas Koskinen, Hubert Scharnagl, Mika Kähönen, Hugh S. Markus, Winfried März, Terho Lehtimäki

**Affiliations:** 1 Department of Clinical Chemistry, Fimlab Laboratories and School of Medicine, University of Tampere, Tampere, Finland; 2 Vth Department of Medicine (Nephrology, Hypertensiology, Endocrinology, Diabetology, Rheumatology), Medical Faculty Mannheim, University of Heidelberg, Heidelberg, Germany; 3 School of Life Science, University of Lincoln, Lincoln, UK; 4 Division of Vascular Surgery, Department of Surgery, Tampere University Hospital, Tampere, Finland; 5 Heart Hospital, Tampere University Hospital, Tampere, Finland; 6 Stroke Prevention Research Unit, Nuffield Department of Clinical Neuroscience, University of Oxford, Oxford, UK; 7 Division of Clinical Neurosciences and Insititute of Genetics and Molecular Medicine, University of Edinburgh, UK; 8 Institute for Stroke and Dementia Research, Klinikum der Universität München, Ludwig-Maximilians-Universität, Munich, Germany & Munich Cluster for Systems Neurology (SyNergy), Munich, Germany; 9 Transplantation Laboratory, Haartman Institute, University of Helsinki, Helsinki, Finland; 10 Heart and Lung Center, Helsinki University Hospital and Helsinki University, Helsinki, Finland; 11 Research Centre of Applied and Preventive Cardiovascular Medicine, University of Turku, Turku, Finland; 12 Satakunta Central Hospital, Department of Surgery, Pori, Finland; 13 Clinical Institute of Medical and Chemical Laboratory Diagnostics, Medical University of Graz, Graz, Austria; 14 Department of Clinical Physiology, Tampere University Hospital and University of Tampere, Tampere, Finland; 15 Clinical Neurosciences, University of Cambridge, Cambridge, UK; 16 Synlab Academy, Synlab Services GmbH, Mannheim, Germany

## Abstract

Asymmetric and symmetric dimethylarginines (ADMA and SDMA) impair nitric oxide bioavailability and have been implicated in the pathogenesis of atrial fibrillation (AF). Alanine–glyoxylate aminotransferase 2 (AGXT2) is the only enzyme capable of metabolizing both of the dimethylarginines. We hypothesized that two functional *AGXT2* missense variants (rs37369, V140I; rs16899974, V498L) are associated with AF and its cardioembolic complications. Association analyses were conducted using 1,834 individulas with AF and 7,159 unaffected individuals from two coronary angiography cohorts and a cohort comprising patients undergoing clinical exercise testing. In coronary angiography patients without structural heart disease, the minor A allele of rs16899974 was associated with any AF (OR = 2.07, 95% CI 1.59-2.68), and with paroxysmal AF (OR = 1.98, 95% CI 1.44–2.74) and chronic AF (OR = 2.03, 95% CI 1.35–3.06) separately. We could not replicate the association with AF in the other two cohorts. However, the A allele of rs16899974 was nominally associated with ischemic stroke risk in the meta-analysis of WTCCC2 ischemic stroke cohorts (3,548 cases, 5,972 controls) and with earlier onset of first-ever ischemic stroke (360 cases) in the cohort of clinical exercise test patients. In conclusion, *AGXT2* variations may be involved in the pathogenesis of AF and its age-related thromboembolic complications.

Circulating asymmetric and symmetric dimethylarginine (ADMA and SDMA) predict mortality in cardiovascular diseases^1^,^2^. Moreover, accumulating epidemiological and experimental evidence have implicated ADMA in the pathogenesis of atrial fibrillation (AF)^3^,^4^,^5^,^6^,^7^, whereas little attention has been paid to the role of SDMA^2^. In patients with a history of AF, the induction of AF during catheter ablation resulted in significantly elevated ADMA levels in the atria and systemic circulation^7^, whereas in another study electrical cardioversion of AF decreased ADMA levels to normal values within 24 hours after AF was terminated^6^. Similarly, higher ADMA levels were associated with the recurrence of AF after electrical cardioversion or catheter ablation in patients with persistent AF^3^,^5^. These data suggests that elevated ADMA levels are not only a consequence of AF but may also be involved in the development of AF by modifying the atrial substrates for AF. Indeed, although only ADMA directly inhibits nitric oxide synthase (NOS), both dimethylarginines can reduce the bioavailability of nitric oxide (NO) by sifting the NOS enzyme from the production of NO to superoxide anion^8^,^9^,^10^, and thus promote oxidative stress, endothelial dysfunction, and inflammation, all of which are associated with atrial remodelling and increased vulnerability to AF^11^,^12^,^13^.

The primary pathway of ADMA deactivation is catalysed by the dimethylarginine dimethylaminohydrolase (DDAH) enzymes, whereas the renal clearance plays a major role in the elimination of SDMA from the systemic circulation. In contrast to DDAH, alanine–glyoxylate aminotransferase 2 (AGXT2), a nuclear-encoded mitochondrial protein, can metabolise not only ADMA but also SDMA in humans^14^,^15^. In addition to a strong expression of AGXT2 in the kidney and liver, *AGXT2 *mRNA expression was recently detected in several other human organs including the heart^16^. As the activity of DDAH is reduced and the generation of ADMA increased via up-regulation of protein arginine methyltransferase 1 (PRMT1) in animal fibrillating atria^4^ also explaining the increased ADMA levels in human AF, it can be hypothesized that the alternative AGXT2-mediated elimination pathway of dimethylarginines could play an important role in the pathogenesis of AF.

We recently fine-mapped the *AGXT2* gene region on chromosome 5p13 for associations between single-nucleotide polymorphisms (SNP) and serum SDMA levels and identified two missense mutations in *AGXT2* (rs37369, p.V140I; rs16899974, p.V498L) to be strongly and independently associated with SDMA levels in a meta-analysis of two large cohorts of European descent^15^. Experimental data indicates that rs37369 modifies the enzyme activity^16^,^17^ whereas an *in silico* analysis showed that the rs16899974 variant may modify the enzyme stability^17^. Therefore, we considered that the two functional *AGXT2* variants may serve as naturally occurring genetic models to study the role of AGXT2 in human AF and its thromboembolic complications, i.e. stroke and its subtypes. More specifically, we hypothesized that these functional variants are (1) associated with paroxysmal and/or chronic AF in patients referred for coronary angiography, (2) associated more strongly with circulating levels of ADMA and SDMA in patients with AF compared to patients in sinus rhythm, and (3) associated with ischemic stroke and its subtypes. As a genetic control, we repeated some of the analyses with a known variant at 4q25 previously associated with AF.

## Results

### Associations of *AGXT2* and 4q25 control variants with atrial fibrillation and its subtypes in patients referred for coronary angiography (LURIC and Corogene)

**Figure 1:**
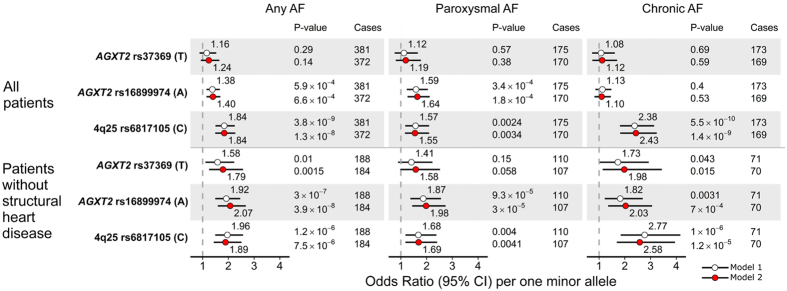
Associations of the AGXT2 and 4q25 variants with prevalent AF and its subtypes in LURIC. ORs per one minor allele increase from logistic regression models assuming additive genetic effect are shown. Model 1: adjusted for age, sex and body mass index. Model 2: Model 1 further adjusted for arterial hypertension, diabetes, coronary artery disease (>50% stenosis), serum NT-proBNP and eGFR. Structural heart disease refers to cardiomyopathy or valvular heart disease.

The clinical characteristics of the study participants for all studies are shown in Supplementary Table 1. In LURIC, 381 (13.0%) patients had prevalent AF at baseline of which 175 (50.3%) and 173 (49.7%) were further classified as paroxysmal or chronic AF, respectively. Those with AF were older (mean age 66.4 versus 62.2 years, P < 0.0001) and had less likely coronary artery disease (56.8% versus 71.6%, P < 0.0001) than patients in sinus rhythm. The associations of *AGXT2* and 4q25 variants with AF and its subtypes are shown in Fig. 1. In the whole study population, rs16899974 showed a significant association with any AF (P = 6.6 × 10^−4^) in a fully adjusted model. Moreover, when analysing paroxysmal and chronic AF cases separately, the association was especially marked with the paroxysmal subtype (P = 1.8 × 10^−4^), whereas there was no association with chronic AF (P = 0.53). We found strong evidence that a history of valvular heart disease attenuated the effect of rs16899974 and rs37369 on AF (p for interaction of 4.3 × 10^−4^ and 0.015, respectively, Table 1). Nominally significant interaction was also observed between rs16899974 and prevalent cardiomyopathy on AF (P = 0.043). When excluding the patients with structural heart disease from the analysis, a highly significant association for *AGXT2* rs16899974 was observed with any AF (P = 3.9 × 10^−8^), and with paroxysmal AF (P = 3.0 × 10^−5^) and chronic AF (P = 7.0 × 10^−4^) separately. In contrast to the *AGXT2* SNPs, the 4q25 variant showed a stronger association with chronic AF than paroxysmal AF in both the whole study population and when excluding the patients with structural heart disease (Fig. 1). When conducting a genome-wide scan using the fully adjusted model in patients without structural heart disease, no additional genome-wide significant associations were identified apart from the signal at the *AGXT2* locus (data not shown).

We sought to replicate the associations of the *AGXT2* variants with AF and its subtypes in an independent cohort. In contrast to LURIC, all subjects in the Corogene study with genotype data available had an acute coronary syndrome (Supplementary Table 1). Of the 2,208 patients included in the analysis, 265 (12.0%) had prevalent AF of which 141 (6.4%) and 107 (4.8%) had paroxysmal and chronic AF, respectively. No associations were observed between *AGXT2* variants and AF or its subtypes in Corogene (all P > 0.05, Supplementary Table 2). However, in accordance with the associations seen in LURIC, the 4q25 variant showed significant associations with any AF (OR = 1.50, 95% CI 1.18–1.91, P = 0.001) and chronic AF (OR = 1.84, 95% CI 1.29–2.61, P = 0.001) but not with paroxysmal AF (OR = 1.25, 95% CI 0.89–1.74, P = 0.20).

### Associations with incident clinical AF and age at AF onset (FINCAVAS)

**Figure 2:**
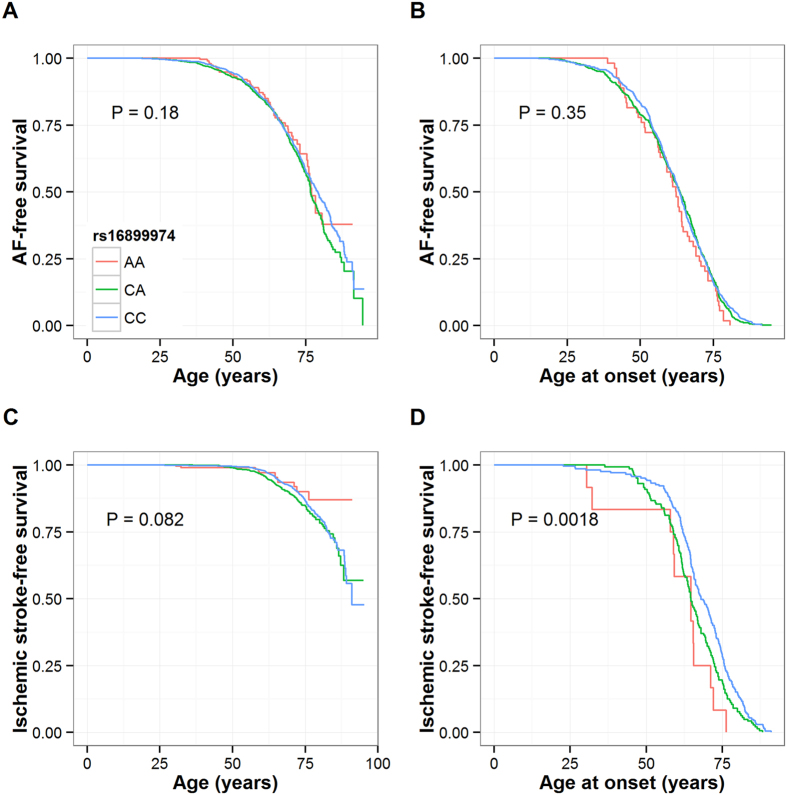
Kaplan-Meier event-free survival for patients participating the FINCAVAS study. Survival curves are shown as a function of the *AGXT2* rs16899974 genotype groups for incident AF (**A**) and ischemic stroke (**C**) and for cases of incident AF (**B**) and ischemic stroke (**D**) only (age of onset analysis).

Of the 3,862 FINCAVAS patients undergoing clinical exercise test between 2001 and 2008, 1,188 (30.8%) had their first AF event diagnosed between 1987 and 2015. The mean (SD) age at the diagnosis was 60.5 (13.3) and 64.6 (13.7) years for men and women, respectively (Supplementary Figure 1). The AF-free survival curves stratified by the rs16899974 genotypes for all patients and AF cases only are displayed in Fig. 2A,B, and for the *AGXT2* rs37369 and 4q25 variants in Supplementary Figures 2 and 3. No associations were seen between the rs16899974 genotypes and incident AF either in the whole study population or in the case-only-analysis (both P > 0.05). As seen in Fig. 2A,B, the survival curves of the three genotype groups start to separate only after around age 75, suggesting that other factors are responsible for clinical AF events in younger patients. In a multivariable analysis, rs16899974 was associated with incident AF and age at AF diagnosis in patients aged ≥75 years at the end of the follow-up, but not in patients aged <75 years at censoring of data (Table 2, Supplementary Table 3). The 4q25 variant showed a strong association in the whole study population; however, rs16899974 appears to be more strongly associated with incident AF in patients aged ≥75 years.

### Associations with history of ischemic stroke and its subtypes (WTCCC2)

We examined whether the two functional *AGXT2* variants are associated with ischemic stroke and its subtypes in the meta-analysis of WTCCC2 ischemic stroke cohorts. As shown in Supplementary Table 4, there was no significant association of rs37369 with all-cause ischemic stroke (IS) whereas rs16899974 showed statistically significant associations with all-cause IS and large-artery atherosclerosis (LAA) stroke subtype with per A allele ORs of 1.04 (95% CI 1.00–1.08, P = 0.032) and 1.10 (95% CI 1.01–1.19, P = 0.021), respectively. Moreover, rs16899974 showed a borderline significant association in the same direction for cardioembolic (CE) stroke with an OR of 1.08 (95% CI 1.00–1.16, P = 0.057). The SNPs were not associated with small-vessel disease (SVD) stroke subtype.

### Associations with incident ischemic stroke and age at first stroke diagnosis (FINCAVAS)

Of the 3,862 FINCAVAS patients undergoing clinical exercise test between 2001 and 2008, 360 (9.3%) had their first ischemic stroke diagnosed between 1987 and 2015. The mean (SD) age at the diagnosis was 65.9 (10.7) and 68.5 (12.4) years for men and women, respectively. Of the 360 incident ischemic stroke cases, 83 (23%) had a previous AF diagnosis. The ischemic stroke-free survival curves stratified by the rs16899974 genotypes for all patients and ischemic stroke cases only are displayed in Fig. 2C,D, and for the *AGXT2* rs37369 and 4q25 variants in Supplementary Figures 2 and 3. None of the three SNPs showed a statistically significant association with incident ischemic stroke. However, rs16899974 was associated with age at onset of a first ischemic stroke both in the univariate analysis (Fig. 2D) and after controlling for sex and previously diagnosed clinical AF (Table 3). Interestingly, this finding was seen only in patients with cryptogenic ischemic stroke (n = 233) with a per minor allele HR of 1.57 (95% CI 1.21–2.04, P = 0.0007) and approximately 3.8 (95% CI 1.5–6.0, P = 0.0009) years earlier age at the diagnosis with each additional copy of the minor allele but not in those with another diagnosis of ischemic stroke (n = 127, both P > 0.05).

### Associations with circulating dimethylarginines in patients with sinus rhythm and AF (LURIC)

**Figure 3:**
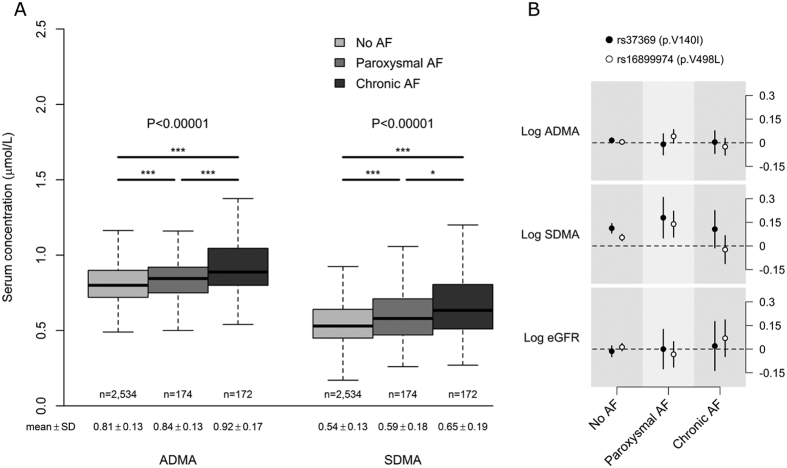
Box plots of untransformed concentrations of ADMA, and SDMA (μmol/l) according to the atrial fibrillation (AF) status in LURIC (**A**). Data are shown as the 25th, 50th, and 75th percentiles (represented by gray boxes), range (shown as whiskers; outliers have been removed), and the median (black horizontal line). *P < 0.05, **P < 0.01, ***P < 0.001. Unadjusted βs for ADMA, SDMA and eGFR by *AGXT2* SNPs and AF status assuming additive genetic effect on the log scale (**B**). Points represent point estimate of the β; vertical bars; the 95% CI.

As displayed in Fig. 3A, there were statistically significant stepwise increases of ADMA and SDMA levels in patients with paroxysmal AF and chronic AF compared to patients in sinus rhythm. The trend across the AF status groups for ADMA remained highly significant after adjusting for age, sex and estimated glomerular filtration rate (eGFR) (p for linear trend = 1.9 × 10^−9^) whereas the association with SDMA was attenuated, but still significant (p for linear trend = 0.0023). The unadjusted associations of the *AGXT2* variants with ADMA, SDMA and eGFR by AF status are shown in Fig. 3B. As we expected, both SNPs showed strong associations with circulating SDMA levels in patients with sinus rhythm. Neither of the *AGXT2* variants was associated with ADMA or eGFR in any of the AF status groups; however, rs16899974 showed a borderline significant association with ADMA in patients with paroxysmal AF (P = 0.057). Moreover, it seems that the effect estimates for SDMA in the paroxysmal AF group are larger than in those with no AF or chronic AF (Fig. 3B). To test this hypothesis the interaction between the AF status groups and the *AGXT2* genotypes on SDMA levels were tested using a two-way ANOVA model with interaction. Statistically significant interaction was observed for rs16899974 in unadjusted model (F = 3.8, P = 0.0044), and when age, sex and eGFR were used as covariates (F = 2.6, P = 0.036). No such statistically significant interactions were observed for rs37369 or when using ADMA as a dependent variable.

## Discussion

In the present study, we analysed the presence of associations of two functional missense *AGXT2* variants with AF and its thromboembolic complications in four independent study cohorts. The p.V498L variant (rs16899974) was associated with an increased risk of both paroxysmal and chronic AF in patients referred for coronary angiography but with no structural heart disease. Moreover, the same A allele of rs16899974 was nominally associated with an increased risk of any ischemic stroke in the meta-analysis of WTCCC2 ischemic stroke cohorts. Finally, rs16899974 was associated with an earlier age at the first ischemic stroke diagnosis in patients undergoing exercise stress testing and with incident clinical AF in patients aged ≥75 years at the time of AF diagnosis in the same cohort.

The two studied *AGXT2* SNPs have not been identified to be associated at a genome-wide significant level with the risk of prevalent or incident AF^18^ or ischemic stroke subtypes^19^ in previous large scale meta-analyses of genome-wide association studies. This is in accordance with the data from the FINCAVAS study showing lack of association with incident AF and ischemic stroke in the whole study population. Furthermore, no associations were observed with prevalent AF or its subtypes in the Corogene study including coronary angiography patients with acute coronary syndrome. In striking contrast with these findings we found a genome-wide significant association of rs16899974 with any AF in patients without structural heart disease in the LURIC study. This association is supported with the observation in FINCAVAS showing that rs16899974 is associated with hospital discharge data based incident clinical AF in patients aged 75 and over at the time of AF diagnosis. Although, there is a separate meta-analysis for lone AF^20^, defined as AF with an onset before 66 years of age in individuals without overt heart disease, no large-scale studies have sought to identify common genetic variants underlying AF risk in the elderly or for paroxysmal and more persistent forms of AF separately.

Interestingly, rs6817105 at 4q25, previously associated with AF in genome-wide association studies, was mainly associated with prevalent chronic AF in both LURIC and Corogene. These associations suggest that the risk allele carriers have a relatively high rate of progression from paroxysmal AF to chronic AF, at least in patients referred for coronary angiography. In contrast, the associations of rs16899974 with both paroxysmal and chronic AF in LURIC, with late onset clinical AF and with earlier age of onset of cryptogenic ischemic stroke suggest a role for AGXT2 in more subclinical forms of AF characterized by a relatively low rate of progression to chronic AF; however, this hypothesis needs to be validated in a prospective setting.

In line with the AF associations in LURIC and FINCAVAS, the AF risk allele of rs16899974 was associated with an earlier onset of cryptogenic ischemic stroke although no association was observed with the ischemic stroke risk in the whole FINCAVAS study population. The association with age at ischemic stroke diagnosis was strengthened after adjusting the analysis for gender and prior AF diagnosis. Moreover, the majority of ischemic stroke cases did not have a prior AF diagnosis and were not having an oral anticoagulation therapy against cardioembolic stroke. The association with the age at ischemic stroke diagnosis could be explained with the observed associations with paroxysmal AF in LURIC and AF in patients aged 75 and over in FINCAVAS. Paroxysmal AF is more likely to be subclinical and asymptomatic than more persistent forms of AF and therefore acute ischemic stroke may be the first clinical manifestation of the underlying subclinical atrial fibrillation. The possible bias due to the underdiagnosed paroxysmal AF cases in FINCAVAS may explain the observation that the association with incident AF is seen only in the elderly with probably more symptoms and more frequent screening for AF than younger patients.

In the LURIC study, circulating ADMA and SDMA levels showed step-wise increases as the AF status was changed from sinus rhythm through paroxysmal and chronic AF independent of age, sex and renal function. In line with these results, a similar step-wise increase in ADMA levels were seen in patients with paroxysmal AF and non-paroxysmal AF compared to patients with no AF in a previous study of coronary angiography patients whereas the association of SDMA with AF was not investigated^21^. More recently, ADMA, but not SDMA, was independently associated with the development of symptomatic AF in patients with acute myocardial infarction^22^. In contrast, SDMA, but not ADMA, was significantly associated with atrial fibrillation in patients with acute ischemic stroke^2^. The lack of association between SDMA and AF in most of the previous studies may reflect the relatively small number of AF cases studied. Moreover, the method of ADMA and SDMA quantification is not standardized complicating comparisons between different studies.

We observed a statistically significant interaction between rs16899974 and AF status groups on circulating SDMA levels independent of age, sex and renal function. Together with the observation that AGXT2 is expressed in the human heart^16^ this interaction suggests a possible role for AGXT2 in the local dimethylarginine metabolism in cardiac tissue; however, due to the observational nature of our study, this interesting hypothesis clearly needs further investigation. Furthermore, the lack of association of rs16899974 with the systemic ADMA levels does not exclude the possibility that AGXT2 could contribute to the ADMA metabolism locally in cardiac tissues. This can be explained by the fact that, unlike SDMA, ADMA is primarily metabolised by the DDAH enzymes which may be able to compensate impaired metabolism by AGXT2 both locally and at the systemic level^23^.

To further support the role of dimethylarginines behind the associations with AF, in a recent large population-based study^24^, SDMA was correlated with markers of atrial remodelling such as left atrial size and atrial conductance time although no independent association was seen between SDMA and AF. These associations are in line with the concept that dimethylarginines could promote the electrical and structural atrial remodeling during AF secondary to oxidative stress and NO synthase uncoupling. It is also possible that the genetic variants of AGXT2 within cardiac tissues alter the NO-related signalling pathways that regulate almost all cardiac ionic channels and modulate the susceptibility to both atrial and ventricular arrhythmias^25^. Interestingly, a recent report showed that ADMA was independently associated with left atrial appendage thrombus in patients with non-valvular atrial fibrillation^26^, further supporting the concept that ADMA-induced endocardial dysfunction could contribute to the increased risk of cardioembolic stroke in AF. Finally, because the AGXT2 enzyme have several substrates other than dimethylarginines^27^ and the systemic levels of dimethylarginines can merely reflect the systemic and/or local activity of AGXT2, our results support further mechanistic studies on the role of dimethylarginines behind the observed associations.

Whether future genome-wide association studies on age at onset of any ischemic or cryptogenic stroke could identify novel genetic variants associated additionally with paroxysmal or subclinical forms of AF is worth investigating because diagnosing of silent AF is challenging. Moreover, as the risk of AF increases substantially with age, an age-at-onset informed^28^ or age stratification-based^29^ approaches could detect novel AF loci whose magnitude of genetic effects differ with age. Whether the *AGXT2* variants could predict the presence of a silent or paroxysmal AF in patients with acute cryptogenic ischemic stroke warrants further investigation. Finally, experimental data for the functional role of rs16899974 is currently lacking.

Some degree of misclassification of AF cases is expected, although this would most likely attenuate the true genetic effect rather than create false positive findings. Because atrial fibrillation is often intermittent and asymptomatic, it is not possible to exclude the possibility that some of those how are classified to have no AF have undetected silent AF. However, this limitation is likely to be present in all clinical data sets in which AF is defined based on hospital discharge diagnostic codes rather than prolonged ambulatory ECG monitoring. In contrast, acute ischemic stroke with neurologic deficits is less likely to be left undetected and without a diagnosis. Moreover, the associations with AF and age at ischemic stroke onset cannot be directly generalized to the general population, other patient groups or individuals of other ancestral backgrounds. Finally, rs16899974 may be in linkage disequilibrium with the causal variant(s) underlying the associations with AF and ischemic stroke phenotypes.

## Conclusions

We found strong evidence that the *AGXT2* p.V498L polymorphism is associated with both paroxysmal and chronic forms of AF in coronary angiographic patients without structural heart disease in ultrasound, and earlier age at onset of ischemic stroke in patients undergoing exercise stress testing. Hence, our study suggests that *AGXT2* variations are involved in the genesis of AF and its age-related thromboembolic complications. Future mechanistic studies should investigate whether these associations are mediated through local metabolism of dimethylarginines by AGXT2 in cardiac tissues.

## Methods

### Study populations

The LURIC study consists of 3,316 Caucasian patients hospitalized for coronary angiography between 1997 and 2000 at a tertiary care center in Southwestern Germany. Clinical indications for angiography were chest pain or a positive non-invasive stress test suggestive of myocardial ischemia. To limit clinical heterogeneity, individuals suffering from acute illnesses other than acute coronary syndrome (ACS), chronic non-cardiac diseases and a history of malignancy within the five past years were excluded.

The Corogene study included 5295 consecutive Finnish patients assigned to coronary angiogram in 4 hospitals servicing 1.5 million people in the Hospital District of Helsinki and Uusimaa. Of the Corogene study, 2500 patients with acute coronary syndrome (ICD-10: I20–I25) were included in a genome-wide association study.

The FINCAVAS study included all consecutive patients referred for a clinically indicated exercise test using a bicycle ergometer at Tampere University Hospital between October 2001 and the end of 2008 and willing to participate. A total of 4,068 participants had a technically successful exercise test. The main indications for the exercise test were suspicion of coronary heart disease (CHD, frequency 46%), evaluation of work capacity (26%), testing vulnerability to arrhythmia during exercise (25%), and adequacy of the CHD treatment (13%); some patients had more than one indication.

Discovery stroke cohorts in WTCCC2 ischemic stroke GWAS included samples from the UK and Germany, with a total of 3,548 cases and 5,972 controls. See further description of the study cohorts and laboratory analyses in Supplementary Methods 1–2 and Supplementary Tables 1, 5 and 6.

This study was conducted according to the Declaration of Helsinki principles and written informed consent was obtained from all participants. The study was performed in accordance with approved guidelines and regulations. The LURIC study was approved by the ethics committee at the Ärztekammer Rheinland-Pfalz. The Corogene study was approved by appropriate Ethics Committees of the Helsinki and Uusimaa Hospital region. The FINCAVAS study was approved by the Ethical Committee of the Hospital District of Pirkanmaa, Finland. For the WTCCC2 ischemic stroke cohorts, the recruitment of patients was approved by the relevant local ethics committees from all the participating centers.

### Genotyping and quality control

In LURIC, genotyping was done by using the Affymetrix Human SNP Array 6.0 at the LURIC Study facility. Genotype imputation was performed using the IMPUTE2 software and the 1000 Genomes March 2012 haplotypes as a reference. Genotyped data was used for rs37369 and imputed data for rs16899974 with an excellent imputation quality (info~0.935).

The Corogene cohort was genotyped with Illumina 660 K BeadChip array at the Sanger Institute (Hixton, Cambridge, UK) and imputed using the 1000 Genomes April 2012 reference panel as a reference.

In FINCAVAS, we genotyped rs16899974 successfully for 3,889 participants using Taqman@SNP Genotyping Assay C__25742181_10 and ABI Prism 7900HT Sequence Detection System (Applied Biosystems, Foster City, CA, USA). No evidence for deviation from the Hardy-Weinberg equilibrium was observed (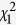 = 0.21, p = 0.65). The data for rs37369 and rs6817105 were obtained for 3,195 individuals by genotyping using the Illumina HumanCardio-Metabo BeadChip or HumanCoreExome chip arrays and imputation using the IMPUTE2 software and 1000 Genomes March 2012 haplotypes as a reference.

For the WTCCC2 samples, Illumina BeadChips were used for genome-wide genotyping and genotype imputation was performed using MACH based on HapMap Phase 2 European (CEU) reference data.

### Classification of atrial fibrillation cases

In LURIC, 2,923 patients had both genotype and atrial fibrillation (AF) status data available. Of the 2,923 participants, 360 had a history of AF at baseline. In addition, 161 individuals were in AF rhythm during the index coronary angiography, of which 21 were not included in the 360 cases diagnosed previously. Therefore, a total of 381 individuals were classified as any AF. Of the 381 AF cases, 348 were further classified as having either paroxysmal AF (n = 175) or chronic AF (n=173).

For the Corogene study, potential prevalent AF cases were screened from the baseline database. For these patients, we ascertained the AF status and its subtype from their medical records. Of the 2,208 patients included in this study, 265, 141 and 107 had any AF, paroxysmal AF and chronic AF, respectively.

For the FINCAVAS participants, clinical AF was ascertained from the central university hospital discharge diagnostic codes (ICD-9-CM 427.3, 427.31, or 427.32; or ICD-10 I48) from 1987 to 2015 (1179 incident AF cases), and study population area-wide electrical ECG recordings from 2005 onward (16 additional incident AF cases). In addition, to further ascertain the AF status at the index exercise stress test, we utilized the data from the baseline examination. According to the FINCAVAS baseline database, 13 patients had a history of AF/flutter or developed one during the exercise stress test but did not have any previous discharge diagnosis of AF with the date of diagnosis and were therefore excluded from all analyses. Both the genotype and phenotype data were available for 1,188 incident AF cases and 2,674 censored controls.

### Classification of ischemic stroke cases

For the WTCCC2 ischemic stroke cohorts, Trial of Org 10172 in Acute Stroke Treatment (TOAST) classification^30^ was performed by an in-house neurologist and all stroke cases were classified into mutually exclusive aetiologic subtypes: large-artery atherosclerosis (LAA), small-vessel disease (SVD), cardioembolic stroke (CE), other aetiology, or unknown aetiology.

For the FINCAVAS cohort, age at the first ischemic stroke was ascertained from the central university hospital discharge diagnostic codes (ICD-9 433.x1, 434 (excluding 434.x0), or 436; or ICD-10 I63.0–I63.9). Of the 3,862 individuals with both genotype and phenotype data available, there were 360 incident ischemic stroke cases diagnosed between 1987 and 2015. In the majority of the cases 233 (65%), the etiology (LAA, SVD or CE) was uncertain at the time of the diagnosis and were therefore diagnosed as a cryptogenic ischemic stroke (I63.9).

### Statistical methods for cross-sectional association and interaction analyses

Statistical analyses were performed under the R statistical environment. The associations of the *AGXT2* and 4q25 variants with AF and its subtypes were tested using multivariable logistic regression analyses assuming an additive genetic model. In addition, we tested the interactions of the studied polymorphisms with established AF risk factors on any AF in LURIC. For the WTCCC2 cohorts, associations of the *AGXT2* variants were tested with unadjusted logistic regression using PLINK^31^ under an additive genetic model and results were meta-analyzed with inverse-variance-weighted method implemented in the METAL software^32^.

### Statistical methods for survival and age of onset analyses

Survival analyses were used to assess associations with incident AF/stroke and differences in age of AF/stroke onset as a function of the studied variants. In addition, for the age of onset analyses, we applied linear regression considering the age of onset as a quantitative trait. Survival curves of time to AF/stroke onset were estimated using the Kaplan–Meier method. We used a Cox regression analysis to examine the effect of the SNPs together with covariates on the survival functions of incident AF and ischemic stroke. We used chronological age as the fundamental time scale in all analyses. All patients were followed to a fixed date (April 2015). Those with no incident AF or ischemic stroke during the observation period were right censored at their last visit at the central hospital or at their last electrical ECG recording in the community, whichever came later. Significance was accepted at P < 0.05 in all analyses.

## Additional Information

**How to cite this article**: Seppälä, I. *et al*. Associations of functional alanine-glyoxylate aminotransferase 2 gene variants with atrial fibrillation and ischemic stroke. *Sci. Rep.* **6**, 23207; doi: 10.1038/srep23207 (2016).

## Supplementary Material

Supplementary Information

## Figures and Tables

**Table 1:** Effect modification of *AGXT2* and 4q25 variants on atrial fibrillation in LURIC.

AF risk factor	Risk factor cases/controls	AF cases/controls	AGXT2 rs37369 (T)			AGXT2 rs16899974 (A)			4q25 rs6817105 (C)		
			OR_int_	(95% CI)	p	OR_int_	(95% CI)	p	OR_int_	(95% CI)	p
Arterial hypertension	1,721/1,202	381/2,542	0.99	(0.56–1.74)	0.98	0.88	(0.60–1.28)	0.49	0.68	(0.46–1.02)	0.065
Diabetes	1,181/1,742	381/2,542	0.81	(0.46–1.40)	0.45	0.82	(0.56–1.19)	0.30	0.91	(0.61–1.36)	0.65
Valvular heart disease	518/2,405	381/2,542	0.45	(0.23–0.86)	0.015	0.46	(0.30–0.71)	0.00043	1.00	(0.63–1.59)	0.99
Aortic stenosis	146/2,405	272/2,279	0.45	(0.09–2.19)	0.32	0.46	(0.18–1.15)	0.096	1.46	(0.57–3.73)	0.43
Aortic insufficiency	80/2,403	269/2,214	0.18	(0.02–1.58)	0.12	0.38	(0.14–1.05)	0.063	1.37	(0.47–4.03)	0.57
Mitral stenosis	16/2,405	260/2,161	0.10	(0.01–1.18)	0.068	0.19	(0.02–1.59)	0.13	0.44	(0.08–2.35)	0.34
Mitral insufficiency	225/2,394	315/2,304	0.36	(0.15–0.88)	0.025	0.48	(0.27–0.85)	0.012	0.88	(0.47–1.63)	0.68
Other	34/2,403	262/2,175	2.66	(0.42–16.9)	0.30	0.83	(0.25–2.76)	0.76	0.39	(0.07–2.01)	0.26
Cardiomyopathy	307/2,616	381/2,542	0.79	(0.37–1.68)	0.54	0.61	(0.38–0.99)	0.043	1.31	(0.75–2.27)	0.34
Ischemic	134/2,612	326/2,420	0.62	(0.21–1.81)	0.38	0.58	(0.29–1.16)	0.12	1.10	(0.52–2.34)	0.79
Dilated	147/2,614	335/2,426	0.76	(0.25–2.26)	0.62	0.46	(0.24–0.90)	0.024	1.34	(0.62–2.91)	0.46
Restricted	24/2,616	296/2,344	1.76	(0.10–32.5)	0.70	2.52	(0.46–13.8)	0.29	0.67	(0.08–5.73)	0.71
Myocardial infarction	1,217/1,706	381/2,542	1.43	(0.81–2.51)	0.21	1.22	(0.82–1.80)	0.33	0.85	(0.55–1.30)	0.46
CVD event (stroke/TIA)	264/2,659	381/2,542	1.27	(0.58–2.78)	0.55	1.53	(0.86–2.74)	0.15	1.18	(0.64–2.18)	0.59

Statistics: Results are from logistic regression analyses adjusted for age, sex and BMI. Interaction effects on a multiplicative scale (OR_int_) per one minor allele increase assuming additive genetic model are shown. OR_int_ = 1 means no interaction on a multiplicative scale.

Notes: In the subgroup analyses of valve disease and cardiomyopathy, controls did not have any valvular disease or cardiomyopathy, respectively.

Abbreviations: CVD, cerebrovascular disease; TIA, transient ischemic attack.

**Table 2:** Associations of *AGXT2* and 4q25 variants with incident clinical AF in FINCAVAS.

Locus	SNP	cases/N	HR	(95% CI)	P
All					
* AGXT2*	rs37369	972/3,122	1.02	(0.87, 1.19)	0.83
* AGXT2*	rs16899974	1188/3,862	1.05	(0.96, 1.16)	0.28
4q25	rs6817105	972/3,122	1.51	(1.36, 1.69)	9.7 × 10^−14^
Age < 75 years					
* AGXT2*	rs37369	802/2,562	0.95	(0.80, 1.12)	0.54
* AGXT2*	rs16899974	991/3,210	0.95	(0.85, 1.05)	0.32
4q25	rs6817105	808/2,587	1.55	(1.37, 1.74)	6.1 × 10^−13^
Age ≥ 75 years					
* AGXT2*	rs37369	170/560	1.07	(0.71, 1.61)	0.75
* AGXT2*	rs16899974	197/662	1.38	(1.10, 1.74)	0.0063
4q25	rs6817105	170/560	1.26	(0.95, 1.67)	0.11

Statistics: HRs per one minor allele increase are from Cox regression models, assuming an additive genetic effect. The baseline hazard function of Cox models are stratified by sex.

Notes: Age is used as the time scale in Cox models. Analyses are stratified based on the age at the end of the follow-up.

**Table 3:** Associations of the *AGXT2* and 4q25 variants with (a) incident ischemic stroke and (b) age at ischemic stroke onset in FINCAVAS.

Locus	SNP	Effect allele	EAF	cases/N	HR	(95% CI)	P
a) incident first ever ischemic stroke							
*AGXT2*	rs37369	T	0.094	295/3,122	0.97	(0.73, 1.30)	0.86
*AGXT2*	rs16899974	A	0.240	360/3,862	1.05	(0.88, 1.24)	0.61
4q25	rs6817105	C	0.156	295/3,122	1.22	(0.90, 1.40)	0.96
b) age at the first ischemic stroke diagnosis							
Linear regression							
Locus	SNP	Effect allele	EAF	N	β	(95% CI)	P
*AGXT2*	rs37369	T	0.086	295	−2.60	(−5.75, 0.55)	0.11
*AGXT2*	rs16899974	A	0.232	360	−3.24	(−5.24, −1.25)	0.0015
4q25	rs6817105	C	0.151	295	−2.60	(−5.14, −0.074)	0.044
Cox model							
Locus	SNP	Effect allele	EAF	N	HR	(95% CI)	P
*AGXT2*	rs37369	T	0.086	295	1.34	(0.99, 1,81)	0.062
*AGXT2*	rs16899974	A	0.232	360	1.44	(1.19, 1.75)	0.00018
4q25	rs6817105	C	0.151	295	1.18	(0.93, 1.51)	0.18

Statistics: HRs and βs (in years) per one minor allele increase are from Cox and linear regression models, respectively, assuming an additive genetic effect. Models are controlled for sex and a history of clinical atrial fibrillation.
