# Supplementary material for: Associations of functional alanine-glyoxylate aminotransferase 2 gene variants with atrial fibrillation and ischemic stroke
Source: Sci Rep. 2016 Mar 17;6:23207. doi: 10.1038/srep23207 (PMC4794714; doi:10.1038/srep23207)
Supplement: Supplementary Information [file srep23207-s1.pdf]

# Supplementary information

## Associations of functional alanine-glyoxylate aminotransferase 2 gene variants with atrial fibrillation and ischemic stroke

Ilkka Seppälä[1], Marcus E. Kleber[2], Steve Bevan[3], Leo-Pekka Lyytikäinen[1], Niku Oksala[1,4], Jussi A. Hernesniemi[1,5], Kari-Matti Mäkelä[1], Peter M Rothwell[6], Cathie Sudlow[7], Martin Dichgans[8], Nina Mononen[1], Efthymia Vlachopoulou[9], Juha Sinisalo[10], Graciela E. Delgado[2], Reijo Laaksonen[1], Tuomas Koskinen[11,12], Hubert Scharnagl[13], Mika Kähönen[14], Hugh S. Markus[15], Winfried März[2,13,16], Terho Lehtimäki[1]

1. Department of Clinical Chemistry, Fimlab Laboratories and School of Medicine, University of Tampere, Tampere, Finland.
2. Vth Department of Medicine (Nephrology, Hypertensiology, Endocrinology, Diabetology, Rheumatology), Medical Faculty Mannheim, University of Heidelberg, Heidelberg, Germany.
3. School of Life Science, University of Lincoln, Lincoln, UK.
4. Division of Vascular Surgery, Department of Surgery, Tampere University Hospital, Tampere, Finland.
5. Heart Hospital, Tampere University Hospital, Tampere, Finland.
6. Stroke Prevention Research Unit, Nuffield Department of Clinical Neuroscience, University of Oxford, Oxford, UK.
7. Division of Clinical Neurosciences and Insititute of Genetics and Molecular Medicine, University of Edinburgh, UK.
8. Institute for Stroke and Dementia Research, Klinikum der Universität München, Ludwig-Maximilians-Universität, Munich, Germany & Munich Cluster for Systems Neurology (SyNergy), Munich, Germany.
9. Transplantation Laboratory, Haartman Institute, University of Helsinki, Helsinki, Finland.
10. Heart and Lung Center, Helsinki University Hospital and Helsinki University, Helsinki, Finland.
11. Research Centre of Applied and Preventive Cardiovascular Medicine, University of Turku, Turku, Finland.
12. Satakunta Central Hospital, Department of Surgery, Pori, Finland.
13. Clinical Institute of Medical and Chemical Laboratory Diagnostics, Medical University of Graz, Graz, Austria.
14. Department of Clinical Physiology, Tampere University Hospital and University of Tampere, Tampere, Finland.
15. Clinical Neurosciences, University of Cambridge, Cambridge, UK.
16. Synlab Academy, Synlab Services GmbH, Mannheim, Germany.

# Supplemental Methods

## Supplementary Methods 1. Study populations and ethics statements.

### *Ludwigshafen Risk and Cardiovascular Health (LURIC) study*

The LURIC study consists of 3,316 Caucasian patients who were referred to coronary angiography because of chest pain at a tertiary care center in Southwest Germany between 1997 and 2000[1]. Inclusion criteria for LURIC were: German ancestry (limitation of genetic heterogeneity), clinical stability (except for acute coronary syndromes) and availability of a coronary angiogram. Exclusion criteria were: any acute illness other than acute coronary syndromes, any chronic disease where non-cardiac disease predominated and a history of malignancy within the last five years. The study was approved by the ethics committee at the Ärztekammer Rheinland-Pfalz and was conducted in accordance with the Declaration of Helsinki. Informed written consent was obtained from all participants. For the 3,034 LURIC participants, all necessary genotype and AF status data were available and they were included in the present study.

### *The Corogene study*

For the COROGENE cohort, initially, all consecutive Finnish patients undergoing coronary angiogram between June, 2006, and March, 2008 (n=5,295), in the Helsinki University Central Hospital were included and a questionnaire, information about previous medical conditions and cardiovascular risk factors, hospital records for patients' history, laboratory measurements, electrocardiogram, echocardiogram, and medication were obtained. Approximately 22% of the patients were angiographically free of coronary artery disease (CAD). Different stages of atherosclerosis were found in 77% of patients, of which 51% had acute coronary syndrome (ACS; n=2,091).[2] Of these patients, 2,085 had acute coronary syndrome (ICD-10: I20–I25) and were included in a genome-wide association study.

### *The Finnish Cardiovascular Study (FINCAVAS)*

All consecutive patients who were referred for an exercise test due to any indication at Tampere University Hospital between October 2001 and December 2008 and who were willing to participate in the Finnish Cardiovascular Study (FINCAVAS) were recruited. A total of 4,068 participants had a technically successful exercise test. The main indications for the exercise test were suspicion of coronary heart disease (CHD, frequency 46%), evaluation of work capacity (26%), testing vulnerability to arrhythmia during exercise (25%), and adequacy of the CHD treatment (13%); some patients had more than one indication. The study protocol has been previously described more extensively[3].

### *Discovery stroke cohorts in WTCCC2 ischaemic stroke*

Discovery stroke cohorts in WTCCC2 ischaemic stroke GWAS included samples from the UK (a-c) and Germany (d), with a total of 3,548 cases and 5,972 controls[4]. Cases were phenotyped and classified into mutually exclusive aetiologic subtypes according to the TOAST classification [5]. The control data set for the British discovery samples was the WTCCC2 common control set, which includes healthy blood donors from the United Kingdom Blood Service's (UKBS) collection and individuals from the 1958 Birth Cohort dataset (58C). The control data set for the German cases was taken from the MONICA/KORA Augsburg Study's population-based controls from the same region in Germany.

(a) St George's Stroke Study, London, UK: Ischaemic stroke patients of European descent attending a cerebrovascular service were recruited in 1995–2008. All cases were phenotyped by one experienced stroke neurologist with a review of original imaging. All patients underwent clinically relevant diagnostic examinations, including brain imaging with computed tomography (CT) and/or magnetic resonance imaging (MRI) as well as ancillary diagnostic investigations including duplex ultrasonography of the carotid and vertebral arteries, echocardiography, Holter monitoring, magnetic resonance angiography (MRA), CT angiography (CTA) and blood tests.

(b) The Oxford Vascular Study, UK: Patients of European descent who had suffered an acute ischaemic stroke or transient ischaemic attack (TIA) with evidence of infarction in brain imaging were recruited during 2002–2008 as a part of a population-based study of all TIA and stroke cases among approximately 91,000 people in Oxfordshire, UK. All cases were phenotyped by one experienced stroke neurologist with a review of original imaging.

(c) The Edinburgh Stroke Study, Scotland, UK: Between 2002 and 2005, consecutive consenting stroke patients who were admitted to or seen as outpatients at the Western General Hospital, Edinburgh, were prospectively recruited. Cases in this study were those with a clinically evident stroke, demonstrated by brain imaging (CT or MRI) to be ischaemic. An experienced stroke physician assessed each patient as soon as possible after the stroke, prospectively recording demographic and clinical details, including vascular risk factors and results of brain imaging and other investigations.

(d) Munich, Germany: White European patients were recruited consecutively from a single dedicated Stroke Unit (Klinikum Großhadern, Ludwig-Maximilians- University of Munich) in 2002–2008. Brain imaging was performed on all patients, with the majority of patients (>80%) undergoing MRI including diffusion-weighted imaging. The diagnosis of ischaemic stroke was based on neurological symptoms in combination with a documented acute infarct visible in neuroimaging. The diagnostic protocol included ECG and duplex ultrasonography of the extracranial arteries in all cases. Transcranial ultrasound, CTA and/or MRA, transthoracic and transesophageal echocardiography as well as Holter monitoring were performed if clinically relevant.

The control data set for the British discovery samples was the WTCCC2 common control set, which includes healthy blood donors from the United Kingdom Blood Service's (UKBS) collection and individuals from the 1958 Birth Cohort dataset (58C). The control data set for the German cases was taken from the MONICA/KORA Augsburg Study's population-based controls from the same region in Germany.

## Supplementary Methods 2. Biochemical measurements.

In LURIC, venous blood samples were drawn after an overnight fast. Routine laboratory measurements were performed as described by Winkelmann et al.[1]. N-terminal pro-B-type natriuretic peptide (NT-pro-BNP) was determined by ElectroChemiLuminescence (ECL) on an Elecsys 2010 (Roche Diagnostics, Mannheim, Germany). ADMA and SDMA were measured from frozen serum (−80 °C) with the reversed-phase HPLC method[6], with slight modifications[7]. Estimated glomerular filtration rate (eGFR) was calculated according to the CKD-EPI study creatinine equation[8].

## Supplementary Methods 3. Assessments of risk factors.

### *LURIC*

Body mass index (BMI) was calculated with the formula: weight (kg) divided by height (in meters) squared. Type 2 diabetes was newly diagnosed using the 2010 criteria of the American Diabetes Association as described previously[9]. Hypertension was diagnosed if the systolic and/or diastolic blood pressure exceeded 140 and/or 90 mmHg or if there was a history of hypertension evident in the use of antihypertensive drugs[1].

Coronary artery disease (CAD) was assessed by means of angiography using the maximum luminal narrowing estimated by visual analysis. CAD was defined as the occurrence of at least one 50% stenosis in at least 1 of 15 coronary segments. MI was defined as evidence of any MI (acute, previous, ST-elevation MI (STEMI) or non-ST-elevation MI (nonSTEMI).

## Supplemental Tables

**Supplementary Table 1. Characteristics of the LURIC, Corogene and FINCAVAS study populations according to AF status at baseline.**

| | LURIC | | | Corogene | | | FINCAVAS | | |
|---|---|---|---|---|---|---|---|---|---|
| | No AF (n=2,542) | AF (n=381) | p | No AF (n=1,943) | AF (n=265) | p | No AF (n=3279) | AF (n=583) | p |
| Age, years | 62.2±10.7 | 66.4±9.0 | <0.0001 | 65.4±11.7 | 73.3±8.41 | <0.0001 | 55.9±13.0 | 60.4±12.4 | <0.0001 |
| Men, % | 1786(70.3%) | 269(70.6%) | 0.94 | 1,363(70.1%) | 171(64.5%) | 0.10 | 1919(58.5%) | 421(72.2%) | <0.0001 |
| Body mass index, kg $m^{-2}$ | 27.4±4.0 | 27.6±4.2 | 0.37 | 27.5±4.7 | 27.7±5.1 | 0.57 | 27.4±4.5 | 28.3±4.5 | <0.0001 |
| Hypertension, % | 1493(58.7%) | 228(59.8%) | 0.72 | 1,036(64.7%) | 134(77.9%) | 0.001 | 1602(49.9%) | 312(54.5%) | 0.0456 |
| Current smokers, % | 503(19.8%) | 55(14.4%) | 0.016 | 614(31.6%) | 36(13.6%) | <0.0001 | 787(24.0%) | 104(17.8%) | 0.0013 |
| Diabetes mellitus, % | 1003(39.5%) | 178(46.7%) | 0.0083 | 383(19.7%) | 74(27.9%) | 0.001 | 396(12.4%) | 93(16.3%) | 0.0114 |
| Coronary artery disease, % | 1799(71.6%) | 213(56.8%) | <0.0001 | 1,894(97.5%) | 252(95.1%) | 0.22 | 979(29.9%) | 145(24.9%) | 0.0167 |
| History of MI, % | 1099(43.2%) | 118(31.0%) | <0.0001 | 1,421(73.1%) | 156(58.9%) | <0.0001 | 631(19.2%) | 92(15.8%) | 0.0552 |
| Systolic BP, mm Hg | 141.4±23.7 | 141.2±23.2 | 0.89 | NA | NA | NA | 136.4±18.8 | 137.3±19.3 | 0.3067 |
| Diastolic BP, mm Hg | 80.9±11.4 | 81.3±11.7 | 0.54 | NA | NA | NA | 80.0±9.7 | 81.2±9.5 | 0.0041 |

Values are means ± standard deviations in cases of continuous variables and percentages in cases of categorical variables.

**Supplementary Table 2. Association of *AGXT2* and 4q25 variants with prevalent AF and its subtypes in Corogene.**

| | Any AF | | | | Paroxysmal AF | | | | Chronic AF | | | |
|---|---|---|---|---|---|---|---|---|---|---|---|---|
| | cases/controls | OR | (95% CI) | p | cases/controls | OR | (95% CI) | p | cases/controls | OR | (95% CI) | p |
| **All patients (n=2,208)** | | | | | | | | | | | | |
| *AGXT2* rs37369 | | | | | | | | | | | | |
| Model 1 | 265/1,943 | 0.872 | (0.63,1.20) | 0.403 | 141/1,943 | 0.914 | (0.59, 1.39) | 0.678 | 107/1,943 | 0.666 | (0.38, 1.14) | 0.138 |
| *AGXT2* rs16899974 | | | | | | | | | | | | |
| Model 1 | 265/1,943 | 0.89 | (0.71, 1.11) | 0.311 | 141/1,943 | 0.839 | (0.61, 1.13) | 0.261 | 107/1,943 | 0.904 | (0.64, 1.27) | 0.564 |
| 4q25 rs6817105 | | | | | | | | | | | | |
| Model 1 | 265/1,943 | 1.497 | (1.18-1.91) | 0.001 | 141/1,943 | 1.245 | (0.89-1.74) | 0.201 | 107/1,943 | 1.838 | (1.29-2.61) | 0.001 |
| **Patients without structural heart disease (valvular heart disease or cardiomyopathy) (n=1,984)** | | | | | | | | | | | | |
| *AGXT2* rs37369 | | | | | | | | | | | | |
| Model 1 | 225/1,759 | 0.843 | (0.58, 1.21) | 0.352 | 117/1,759 | 0.832 | (0.51, 1.36) | 0.463 | 91/1,759 | 0.654 | (0.35, 1.19) | 0.168 |
| *AGXT2* rs16899974 | | | | | | | | | | | | |
| Model 1 | 225/1,759 | 0.841 | (0.65, 1.07) | 0.169 | 117/1,759 | 0.833 | (0.59, 1.16) | 0.284 | 91/1,759 | 0.782 | (0.53, 1.14) | 0.21 |
| 4q25 rs6817105 | | | | | | | | | | | | |
| Model 1 | 225/1,759 | 1.643 | (1.27-2.12) | 0.0001 | 117/1,759 | 1.401 | (0.99-1.99) | 0.061 | 91/1,759 | 1.979 | (1.37-2.86) | 0.0003 |

**Statistics:** ORs per one minor allele increase from logistic regression models assuming additive genetic effect are shown.

Model 1: adjusted for age, sex and body mass index.

**Supplementary Table 3. Associations of *AGXT2* and 4q25 variants with age at AF diagnosis in FINCAVAS.**

| | | All | | | | Age<75 years | | | | Age≥75 years | | | |
|---|---|---|---|---|---|---|---|---|---|---|---|---|---|
| **Linear regression** | | | | | | | | | | | | | |
| Locus | SNP | N | β | (95% CI) | P | N | β | (95% CI) | P | N | β | (95% CI) | P |
| *AGXT2* | rs37369 | 972 | -0.59 | (-2.64, 1.47) | 0.58 | 802 | -0.17 | (-2.10, 1.77) | 0.87 | 170 | -0.78 | (-2.37, 0.81) | 0.33 |
| *AGXT2* | rs16899974 | 1188 | -0.54 | (-1.85, 0.76) | 0.41 | 991 | -0.55 | (-1.80, 0.70) | 0.39 | 197 | -1.45 | (-2.41, -0.49) | 0.0032 |
| 4q25 | rs6817105 | 972 | -3.53 | (-4.99, -2.06) | $2.5\times10^{-6}$ | 802 | -2.44 | (-3.81, -1.07) | $4.9\times10^{-4}$ | 170 | -1.19 | (-2.42, 0.041) | 0.058 |
| **Cox model** | | | | | | | | | | | | | |
| Locus | SNP | N | HR | (95% CI) | P | N | HR | (95% CI) | P | N | HR | (95% CI) | P |
| *AGXT2* | rs37369 | 972 | 1.08 | (0.92, 1.26) | 0.36 | 802 | 1.01 | (0.85, 1.20) | 0.88 | 170 | 1.23 | (0.81, 1.85) | 0.33 |
| *AGXT2* | rs16899974 | 1188 | 1.07 | (0.97, 1.18) | 0.18 | 991 | 1.05 | (0.94, 1.17) | 0.36 | 197 | 1.53 | (1.18, 1.99) | 0.0013 |
| 4q25 | rs6817105 | 972 | 1.30 | (1.17, 1.45) | $2.2\times10^{-6}$ | 802 | 1.19 | (1.06, 1.33) | 0.0039 | 170 | 1.34 | (1.01, 1.77) | 0.041 |

**Statistics:** HRs and βs (in years) per one minor allele increase are from Cox and linear regression models, respectively, assuming an additive genetic effect. The baseline hazard function of Cox models are stratified by sex and linear regression models are adjusted for sex.

**Notes:** Age is used as the time scale in Cox models. Analyses are stratified based on the age at the end of the follow-up.

**Supplementary Table 4. Association of the *AGXT2* SNPs with ischemic stroke and its subtypes in the WTCCC2 ischemic stroke cohorts.**

| SNP | A1/A2 | EAF (%) | Effect direction | Association | | Heterogeneity | |
|---|---|---|---|---|---|---|---|
| | | | | OR (95% CI) | p | Q (p) | $I^2$ |
| All ischemic stroke | | | | | | | |
| rs37369 | A/G | 9.03 | -+-++++-++-+++ | 1.04 (0.99-1.10) | 0.11 | 12.6 (0.56) | 0 |
| rs16899974 | T/G | 22.7 | ++-++-+--+--++- | 1.04 (1.00-1.08) | 0.032 | 17.6 (0.22) | 9.3 |
| Cardioembolic stroke | | | | | | | |
| rs37369 | A/G | 9.18 | -+--+---+-+-+ | 1.01 (0.91-1.13) | 0.81 | 4.45 (0.97) | 0 |
| rs16899974 | T/G | 22.3 | +-+++-+----++ | 1.08 (1.00-1.16) | 0.057 | 8.69 (0.73) | 0 |
| Large vessel stroke | | | | | | | |
| rs37369 | A/G | 9.11 | ---++-+--++ | 1.04 (0.92-1.17) | 0.51 | 11.4 (0.33) | -5.4 |
| rs16899974 | T/G | 22.7 | -+--+-+++++ | 1.10 (1.01-1.19) | 0.021 | 13.3 (0.21) | 9.8 |
| Small vessel stroke | | | | | | | |
| rs37369 | A/G | 8.86 | ++---+--+-++ | 1.08 (0.96-1.22) | 0.18 | 10.7 (0.47) | 0 |
| rs16899974 | T/G | 22.8 | ++--++---+++ | 1.02 (0.94-1.11) | 0.62 | 11.4 (0.41) | -13.8 |

A1 (Effect Allele), A2 (Non Effect Allele), EAF (Effect Allele Frequency), Q (Chi square statistics), $I^2$ (Index test quantifies extent of variation across studies in a meta-analysis).

**Supplementary Table 5. Characteristics of the FINCAVAS study population by atrial fibrillation (AF) and ischemic stroke (IS) status at the end of the follow-up.**

| | No AF (n=2,674) | AF (n=1,188) | p | No IS (n=3,502) | IS (n=360) | p |
|---|---|---|---|---|---|---|
| Age, years | 62.5±13.8 | 61.8±13.5 | 0.15 | 63.8±13.8 | 66.8±11.3 | <0.0001 |
| Men, % | 1526 (57.1%) | 814 (68.5%) | <0.0001 | 2102 (60.0%) | 238 (66.1%) | 0.028 |
| Hypertension, % | 811 (30.3%) | 287 (24.2%) | 0.0001 | 1141 (32.6%) | 133 (36.9%) | 0.11 |
| Diabetes mellitus, % | 799 (29.9%) | 263 (22.1%) | <0.0001 | 1110 (31.7%) | 128 (35.6%) | 0.15 |
| Coronary artery disease, % | 965 (36.1%) | 280 (23.6%) | <0.0001 | 1431 (40.9%) | 107 (29.7%) | 0.0001 |
| History of MI, % | 623 (23.3%) | 225 (18.9%) | 0.0029 | 790 (22.6%) | 104 (28.9%) | 0.0081 |
| Peripheral artery disease, % | 111 (4.2%) | 41 (3.5%) | 0.35 | 162 (4.6%) | 16 (4.4%) | 0.98 |
| Heart failure, % | 194 (7.3%) | 150 (12.6%) | <0.0001 | 450 (12.8%) | 47 (13.1%) | 0.98 |
| Atrial fibrillation, % | - | - | - | 1010 (28.8%) | 100 (27.8%) | 0.72 |
| Ischemic stroke, % | 183 (6.8%) | 94 (7.9%) | 0.26 | - | - | - |
| Transient ischemic attack (TIA), % | 93 (3.5%) | 27 (2.3%) | 0.059 | 100 (2.9%) | 30 (8.3%) | <0.0001 |
| Any valvular heart disease, % | 194 (7.3%) | 141 (11.9%) | <0.0001 | 398 (11.4%) | 33 (9.2%) | 0.24 |
| Aortic stenosis, % | 69 (2.6%) | 43 (3.6%) | 0.095 | 143 (4.1%) | 13 (3.6%) | 0.77 |
| Aortic regurgitation | 121 (4.5%) | 77 (6.5%) | 0.014 | 249 (7.1%) | 21 (5.8%) | 0.43 |
| Mitral regurgitation | 61 (2.3%) | 54 (4.5%) | 0.0002 | 171 (4.9%) | 8 (2.2%) | 0.031 |
| Any cardiomyopathy, % | 132 (4.9%) | 64 (5.4%) | 0.61 | 233 (6.7%) | 11 (3.1%) | 0.011 |

Values are means ± standard deviations in cases of continuous variables and percentages in cases of categorical variables.

**Supplementary Table 6. The general characteristics of ischaemic stroke cases and controls in the WTCCC2 study populations.**

| Cohort | All cases | Subtypes | | | Controls |
|---|---|---|---|---|---|
| | | CE | LAA | SVD | |
| | | | **Number** | | |
| WTCCC2-Germany | 1,174 | 330 | 346 | 106 | 797 |
| WTCCC2-UK | 2,374 | 460 | 498 | 474 | 5,175 |
| *Total* | *3,548* | *790* | *844* | *580* | *5,972* |
| | | | **Age (mean ± SD)** | | |
| WTCCC2-Germany | 66.9 ± 12.9 | 71.7 ± 12.1 | 65.8 ± 10.8 | 65.9 ± 11.4 | 62.7 ± 10.9 |
| WTCCC2-UK | 72.2 ± 12.3 | 77.0 ± 12.9 | 70.0 ± 10.2 | 70.4 ± 11.7 | 52* |
| | | | **Male sex (%)** | | |
| WTCCC2-Germany | 61.9 | 52.1 | 70.7 | 72.6 | 51.4 |
| WTCCC2-UK | 53.7 | 59.4 | 63.3 | 49.8 | 50.5 |

**Abbreviations:** WTCCC2, Wellcome Trust Case-control consortium II; IS, all ischaemic stroke cases; SVD, small-vessel disease; CE, cardioembolic source; LAA, large-artery atherosclerosis. *A subset of controls from WTCCC2-UK were from the 1958 birth cohort; the age of all other participants was unknown.

## Supplemental Figures

**Supplementary Figure 1. Kaplan-Meier event-free survival as a function of gender for patients participating the FINCAVAS study.**

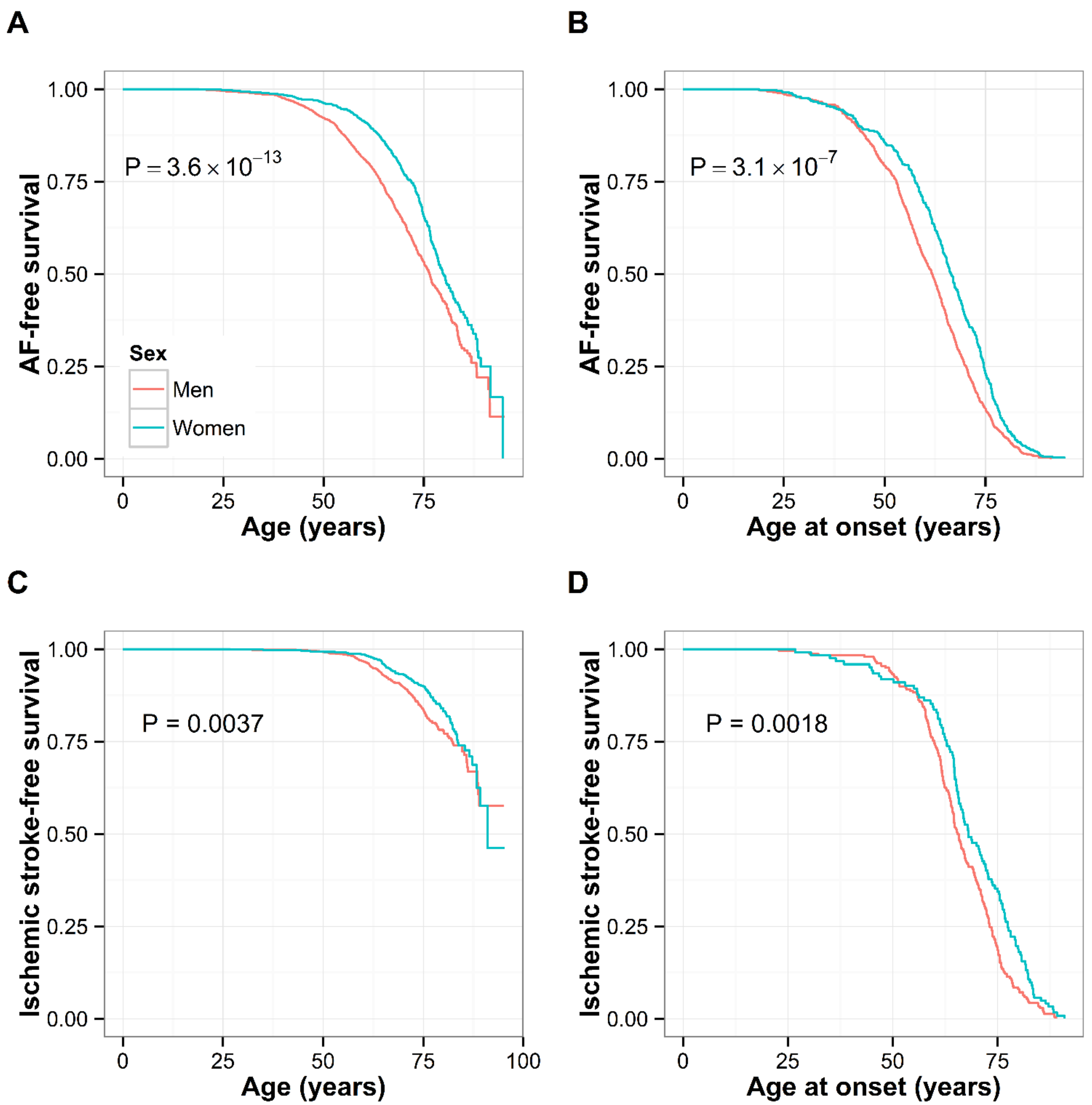

**Supplementary Figure 2. Kaplan-Meier AF-free survival as a function of rs37369 genotypes for patients participating the FINCAVAS study.**

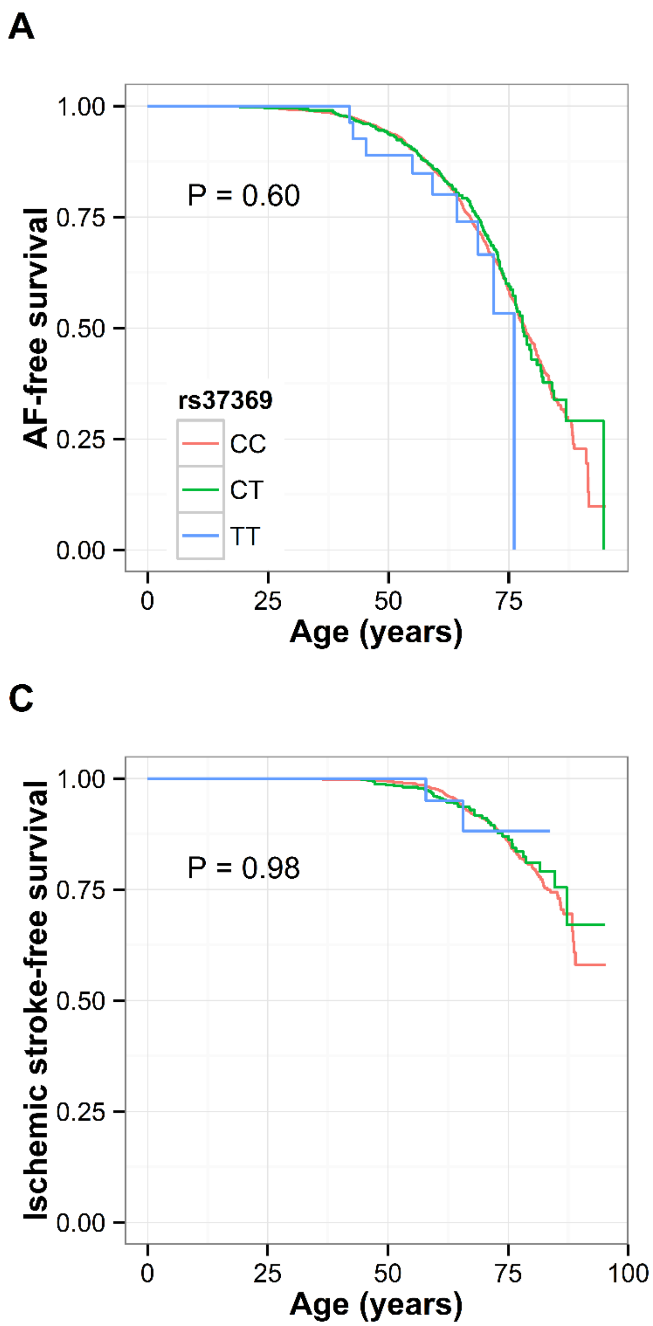

**Supplementary Figure 3. Kaplan-Meier event-free survival as a function of rs6817105 genotypes for patients participating the FINCAVAS study.**

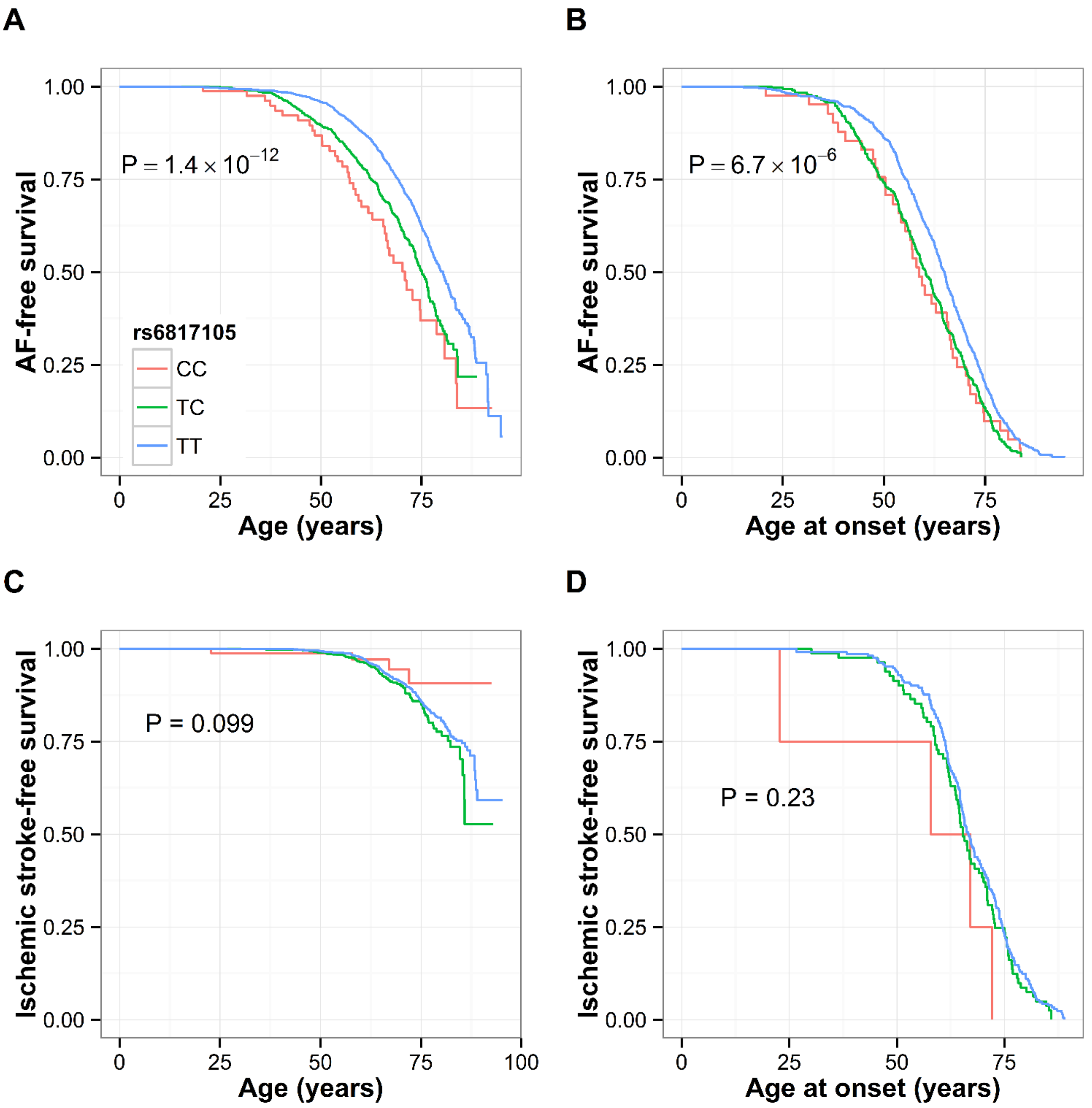

## Supplemental References


1. Winkelmann, B. R. *et al*. Rationale and design of the LURIC study--a resource for functional genomics, pharmacogenomics and long-term prognosis of cardiovascular disease. *Pharmacogenomics* **2**, S1-73 (2001).

2. Vaara, S. *et al*. Cohort Profile: the Corogene study. *Int. J. Epidemiol.* **41**, 1265-1271 (2012).

3. Nieminen, T. *et al*. The Finnish Cardiovascular Study (FINCAVAS): characterising patients with high risk of cardiovascular morbidity and mortality. *BMC Cardiovasc. Disord.* **6**, 9 (2006).

4. International Stroke Genetics Consortium (ISGC) *et al*. Genome-wide association study identifies a variant in HDAC9 associated with large vessel ischemic stroke. *Nat. Genet.* **44**, 328-333 (2012).

5. Adams, H. P.,Jr *et al*. Classification of subtype of acute ischemic stroke. Definitions for use in a multicenter clinical trial. TOAST. Trial of Org 10172 in Acute Stroke Treatment. *Stroke* **24**, 35-41 (1993).

6. Teerlink, T., Nijveldt, R. J., de Jong, S. & van Leeuwen, P. A. Determination of arginine, asymmetric dimethylarginine, and symmetric dimethylarginine in human plasma and other biological samples by high-performance liquid chromatography. *Anal. Biochem.* **303**, 131-137 (2002).

7. Meinitzer, A. *et al*. Reference values for plasma concentrations of asymmetrical dimethylarginine (ADMA) and other arginine metabolites in men after validation of a chromatographic method. *Clin. Chim. Acta* **384**, 141-148 (2007).

8. Levey, A. S. *et al*. A new equation to estimate glomerular filtration rate. *Ann. Intern. Med.* **150**, 604-612 (2009).

9. Silbernagel, G. *et al*. Duration of type 2 diabetes strongly predicts all-cause and cardiovascular mortality in people referred for coronary angiography. *Atherosclerosis* (2012).
